# Dermatological manifestations of hematologic neoplasms. Part II: nonspecific skin lesions/paraneoplastic diseases^☆^

**DOI:** 10.1016/j.abd.2022.08.005

**Published:** 2023-01-20

**Authors:** Patricia Karla de Souza, Rafael Oliveira Amorim, Letícia Siqueira Sousa, Mariana Dias Batista

**Affiliations:** a Hospital Israelita Albert Einstein, São Paulo, SP, Brazil; b Department of Dermatology, Universidade Federal de São Paulo, São Paulo, SP, Brazil

**Keywords:** Erythema nodosum, Pruritus, Pyoderma gangrenosum, Sweet syndrome, Vasculitis

## Abstract

Cutaneous manifestations occur in the course of hematologic malignancies and precede, accompany or occur late in relation to the diagnosis. They result from paraneoplastic phenomena, tumor infiltrations, immunosuppression resulting from the hematologic disease itself or its treatment. The dermatologist must be aware of these conditions that may be helpful both in the diagnosis of the underlying disease and in reducing patient morbidity. This review (part II) addresses the paraneoplastic dermatological changes associated with systemic hematologic malignancies.

## Introduction

This article, part II of the review on the dermatological manifestations of hematologic neoplasms, addresses the nonspecific (or paraneoplastic) dermatoses of systemic hematologic neoplasms. The main dermatological conditions in this context will be discussed.

Paraneoplastic dermatosis is defined as a group of skin diseases that have a strong association with the presence of internal malignancy; they precede, accompany, or occur late in relation to the diagnosis of the neoplasm, without the presence of cancer cells in the skin.^1^, ^2^ Some dermatoses occur only in the presence of a neoplasm and are called obligate paraneoplastic dermatoses, while others are associated with other conditions, being called facultative paraneoplastic dermatoses.^2^

Table 1 summarizes the paraneoplastic dermatological manifestations, the hematologic neoplasms associated with these manifestations, and possible treatments. Table 2 shows the frequencies of these dermatoses according to literature data.

**Table 1:** Nonspecific cutaneous manifestations of hematologic malignancies: associations and management.

Dermatological diagnosis	Most frequently found hematologic alteration	Typical dermatological manifestation	Management
**Sweet syndrome**	AML, chronic myeloid neoplasms, AMML Rarely: monoclonal gammopathies, MM, lymphoid neoplasms	Erythematous or violaceous and painful nodules, papules, and plaques	Systemic corticoids Potassium iodide Dapsone Colchicine

**Pyoderma gangrenosum**	AML	Classic form: painful ulcer with irregular, undermined violaceous borders, sterile inflammatory exudate and necrotic base	Local wound care – zinc oxide or petrolatum on wound borders
	Others: MDS, CML, PV, essential thrombocythemia, myelofibrosis		
		Bullous form: bullae with blue-gray borders progressing to shallow erosion/ulcers – face and arms	Mild cases: High-potency topical corticosteroids or calcineurin inhibitors; intralesional corticosteroids
		Vegetans form: Erythematous, exophytic, and verrucous lesions – head and neck	Extensive cases: Systemic corticosteroid; Ciclosporin
		Pustular form: painful pustules on an erythematous base	Others: pulse therapy with methylprednisolone, methotrexate, mycophenolate, colchicine, sulfasalazine, dapsone, minocycline, apremilast, thalidomide
			Immunobiologicals: infliximab, adalimumab, etanercept, ustekinumab
			Refractory disease: intravenous immunoglobulin, cyclophosphamide, chlorambucil
**Subcorneal pustulosis**	IgA paraproteinemia	Pustules with serpiginous distribution ‒ trunk and intertriginous areas	Dapsone
	Others: IgA myeloma, aplastic anemia, lymphomas, CML, PV		Phototherapy, topical corticosteroids, systemic corticosteroids, and acitretin
		Larger pustules: secretion collected in the lower portion (half and half).	
**Neutrophilic eccrine hidradenitis**	AML and CML with or without chemotherapy	Disseminated erythematous, edematous, pruriginous, or painful plaques ‒ hands, face, extremities	Systemic corticoid
	Others: LLC B, myelomonocytic leukemia, ESL and non-Hodgkin's lymphoma		Dapsone
**Eosinophilic dermatosis**	LLC B	Pruriginous papules, vesicles, bullae or nodules.	Topical corticoid
	Others: B-cell lymphoproliferative diseases, acute T-lymphoma and leukemia		Phototherapy
		Similar to insect bite reaction.	Systemic corticoid
			Dapsone
			Immunosuppressants: methotrexate, azathioprine, lenalidomide, dupilumab
**Pruritus**	Myeloproliferative neoplasms (CML, PV, primary myelofibrosis), essential thrombocytosis	Most common: Aquagenic pruritus – an itching, stinging, burning, or prickling sensation after contact with water on the skin	Serotonin reuptake inhibitors – paroxetine
			Anticonvulsants - gabapentin, pregabalin
	Hodgkin's and non-Hodgkin's lymphomas		Opioid receptor antagonist – Naltrexone
			Phototherapy
			Acetylsalicylic acid in PV
			Neurokinin-1 receptor antagonist: aprepitant
			Corticosteroids in intractable cases
			Thalidomide in patients receiving palliative care
**Cutaneous small vessel vasculitis**	MDS	Painful/pruriginous palpable purpura	Treatment of the underlying disease
	Others: AML, CML, myelofibrosis, PV, essential thrombocythemia		
		Mainly: distal lower limbs	Systemic corticoid
**Polyarteritis nodosa**	Hairy cell leukemia	Subcutaneous nodules, palpable purpura, livedo reticularis or racemosa, ulcerations and bullae	High-dose systemic corticosteroids
		Mainly: lower limbs	Immunosuppressants: methotrexate, cyclophosphamide
**Erythema elevatum diutinum**	IgA type monoclonal gammopathies	Erythematous violaceous papules, nodules, or plaques ‒ extensor surfaces of limbs	Dapsone
			Localized cases: intralesional corticosteroid, surgical excision
	MDS		
**Paraneoplastic pemphigus**	Non-Hodgkin’s lymphoma CLL Castleman’s disease Waldenström’s macroglobulinemia	Erosions progressing to severe oral and conjunctival ulcerations – entire oropharyngeal surface/lip vermilion Severe membranous pseudoconjunctivitis	Prednisone Ciclosporin Cyclophosphamide Other agents: rituximab, alemtuzumab
**Acquired ichthyosis**	Hodgkin’s lymphoma	Hypochromic/gray rhomboid scales with lamellar roughness/desquamation.	Skin hydration Keratolytic agents
	Others: Non-Hodgkin lymphoma, cutaneous T-cell lymphoma, leiomyosarcoma, MF, MM	Similar to fish scales	Treatment of the underlying disease
		Trunk and limbs – extensor surfaces, sparing flexures	
**Erythema nodosum**	LMA, LMC, LMMC	Tender, erythematous nodules and plaques 1–6 cm in diameter	Compression bandages and limb elevation
		Symmetrical lesions in lower/pre-tibial distal extremities	NSAIDs
			Systemic corticosteroid, potassium iodide, colchicine, dapsone, hydroxychloroquine
**Diffuse plane xanthoma**	MM	Irregular, diffuse, symmetrical, asymptomatic yellow-orange macules or plaques	Remission of the haematological condition results in cutaneous improvement
	Monoclonal gammopathies		
		Predilection for face, trunk and intertriginous areas	
**Scleromyxedema**	MM	Generalized eruption of firm, waxy, 2–3 mm, cupuliform/flat papules – hands, forearms, head, neck, trunk, and upper thighs	First line: Intravenous immunoglobulin
	Other: lymphomas, Waldenström’s macroglobulinemia and AMML		Thalidomide (or lenalidomide)
			Systemic corticosteroid
		Linear distribution with surrounding shiny and hardened skin (sclerodermoid)	Extracorporeal photochemotherapy
			PUVA
			Electron beam, topical corticosteroids/retinoids
**Necrobiotic xanthogranuloma**	Monoclonal gammopathy and multiple myeloma	Multiple, asymptomatic, yellowish to reddish-brown indurated papules and nodules. Slow evolution to large plaques.	Topical and systemic corticosteroids, thalidomide, high-dose intravenous immunoglobulin (IVIG), chlorambucil, among others
	Others: Non-Hodgkin's and Hodgkin's lymphoma, Waldenström’s macroglobulinemia, MDS, CLL		

**Prurigo**	Leukemia and Hodgkin's disease	Symmetrically distributed papules, hyperkeratotic or excoriated nodules, and scars.	Diagnosis and treatment of the underlying disease
		Lower limbs and trunk – areas accessible to scratching	Topical corticosteroids, phototherapy and antihistamines – little effective

**Table 2:** Epidemiological data of nonspecific cutaneous manifestations of hematologic malignancies.

Manifestation	Association with hematologic diseases
**Sweet syndrome**	20% related to hematologic malignancies
	55% in the histiocytic form
**Pyoderma gangrenosum**	3,9‒7% related to hematologic malignancies
**Subcorneal pustulosis**	Unknown prevalence
**Neutrophilic eccrine hidradenitis**	Unknown prevalence
	67% associated to AML cases that underwent chemotherapy
**Eosinophilic dermatosis**	Paraneoplastic dermatosis mainly associated with CLL B
**Pruritus**	2% related to hematologic malignancies
**Cutaneous small vessel vasculitis**	3,8–8% related to hematologic malignancies
**Poliarterite nodosa**	Unknown prevalence
**Erythema elevatum diutinum**	Unknown prevalence
	16%–42% – underlying hematologic abnormality
**Paraneoplastic pemphigus**	84% related to hematologic malignancies
**Acquired ichthyosis**	Unknown prevalence
**Erythema nodosum**	0%–4% related to hematologic malignancies in case reviews in the literature
**Diffuse plane xanthoma**	48% in case reviews in the literature
**Scleromyxedema**	Monoclonal gammopathy is one of the diagnostic criteria, and it is atypical not to be found
**Necrobiotic xanthogranuloma**	80% of cases: associated monoclonal gammopathy
	10% develop into MM

## Nonspecific skin lesions related to systemic hematologic malignancy/Paraneoplastic dermatoses

### Neutrophilic dermatoses

Neutrophilic dermatoses consist of a group of cutaneous manifestations characterized by polymorphic lesions, manifested on histopathology by a dermal polymorphonuclear infiltrate.^3^ They include Sweet syndrome, pyoderma gangrenosum, subcorneal pustulosis, and neutrophilic eccrine hidradenitis.^3^, ^4^ Transition or overlap lesions between clinical forms have also been described, as well as extracutaneous involvement.^5^

The pathophysiological mechanisms implicated in its development include increased expression of proinflammatory cytokines responsible for the recruitment and migration of neutrophils to the skin, such as IL-1, IL-8, IL-17 and TNF-α.^6^ In the pathogenesis, genetic factors are also implicated: patients with neutrophilic dermatoses have mutations that also occur in autoinflammatory diseases, in addition to mutations in genes that regulate innate immunity.^6^

Three clinicopathological types of neutrophilic dermatoses have been described: the deep or hypodermic type, which includes pyoderma gangrenosum; plaque or dermal type, which includes Sweet syndrome, and superficial or epidermal form, which includes subcorneal pustulosis.^7^

Neutrophilic dermatoses are classically associated with hematologic malignancies and occur concomitantly with the diagnosis, before or after. They are most often associated with myeloid malignancies, such as acute myeloid leukemia (AML), chronic myeloid leukemia (CML), chronic myelomonocytic leukemia (CMML), and more rarely associated with monoclonal gammopathies, multiple myeloma (MM), and lymphoid neoplasms.^8^ Specifically, in the case of myeloid neoplasms, there is evidence that the neutrophils that infiltrate the skin are clones related to the neoplastic cells.^9^

Some drugs used in the treatment of hematologic malignancies may induce the development of neutrophilic dermatoses, such as azacitidine, imatinib, lenalidomide, and granulocyte colony-stimulating factors.^6^ Neutrophilic dermatoses can also occur as a cutaneous adverse event to checkpoint inhibitors.^10^

### Sweet Syndrome

**Figure 1:**
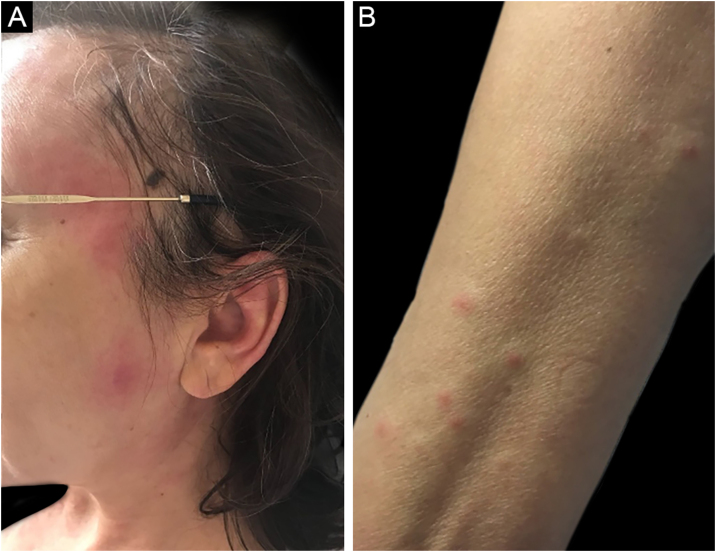
Sweet syndrome in a patient with AML secondary to MDS. Erythematous-edematous papules and plaques affecting (A), face and (B), anterior region of the forearm.

Sweet syndrome or acute febrile neutrophilic dermatosis is an inflammatory syndrome characterized by the abrupt onset of painful, erythematous or violaceous nodules, papules, and plaques (Fig. 1) accompanied by fever and leukocytosis.^3^

Clinical subcutaneous forms similar to extensive cellulitis have been previously described and are frequently associated with hematologic malignancies, while bullous and ulcerated forms also occur in idiopathic Sweet syndrome.^6^ Extracutaneous findings include arthralgia or arthritis, ocular involvement, alveolitis, hepatitis, myositis, aseptic meningitis, and gastrointestinal involvement.^4^ The phenomenon of pathergy can occur and 20% of patients have associated pruritus.^11^ It is recurrent in approximately one-third of the cases.^7^

Three variants have been described: idiopathic or classic, drug-related, and associated with malignancies. Sweet syndrome associated with malignancies accounts for 20% of all cases.^12^

Its pathogenesis involves hypersensitivity reaction to tumor antigens, or changes in the cytokine profile, with high levels of IL-4, IL-6, interferon-gamma, granulocyte colony-stimulating factor, or genetic susceptibility factors.^6^

The diagnosis depends on the presence of clinical criteria and histopathological examination, which demonstrates a dermal neutrophilic infiltrate without vasculitis. There may also be leukocytoclasia and endothelial edema.^13^

An infrequent variant of Sweet syndrome, also associated with hematologic malignancies, is histiocytoid Sweet syndrome. Its clinical aspect is similar to that of classic Sweet syndrome but shows a dermal infiltrate of myelomonocytic cells with histiocytoid morphology.^14^ Up to 55% of cases of histiocytoid Sweet syndrome are associated with malignancies, among which myelodysplastic syndrome stands out.^15^

Treatment is typically carried out with systemic corticosteroids at a dose of 0.5 to 1 mg/kg of prednisone. Alternatives for cases with contraindications to the use of corticosteroids include potassium iodide (300 mg, 3 times/day); dapsone, at a target dose of 100 to 200 mg/day, and colchicine, at a dose of 1 to 1.5 mg/day.^16^

### Pyoderma gangrenosum

Pyoderma gangrenosum is an uncommon neutrophilic dermatosis that begins as a painful papule or pustule, progresses to a nodule, and subsequently progresses to ulceration.^3^ It is typically a diagnosis of exclusion after other infectious or vascular causes of ulcers have been ruled out.^17^

In the pathogenesis of pyoderma gangrenosum, an increase in cytokines and neutrophil chemotactic factors, such as IL-1β, IL-17, TNF-α, IL-8, IL-6, IL-17 and IL-23, has been previously demonstrated. Additionally, there is increased expression of matrix metalloproteinases (MMPs), especially MMPs 9 and 10.^18^

The clinical picture, in the classic form, is characterized by ulcers with irregular, undermined violaceous borders, sterile inflammatory exudate, and a necrotic base. The lesions are typically painful, and the pain is more intense during periods of lesion progression.^7^ The phenomenon of pathergy has been described in 31% of cases,^19^ and lesions were triggered in up to 15% of cases in patients with a previous history of pyoderma gangrenosum submitted to a surgical procedure.^20^ After the condition is resolved, cribriform scars occur.^6^

**Figure 2:**
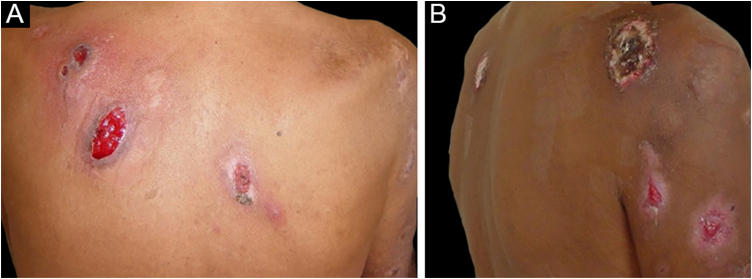
Bullous pyoderma gangrenosum. Shallow ulcers with blue-gray borders (A) on the back and (B) on the upper limb.

Three other clinical forms have been described: bullous, pustular and vegetans or granulomatous. The three forms have in common with the classic form the onset of the lesion as a papule or pustule, which develops into a nodule. In the bullous form (Fig. 2), the lesions occur in the arms or face and are characterized by bullae with a grayish-blue border that progresses into erosions and the formation of shallow ulcers. The pustular form presents with painful pustules on an erythematous base and may be accompanied by fever and arthralgia. The vegetans or granulomatous form occurs preferentially in the head and neck and is characterized by ulcers that gradually transform into erythematous exophytic lesions with a verrucous surface.^7^, ^17^ The periostomal form occurs in association with inflammatory bowel diseases and may be exacerbated by the phenomenon of pathergy.^21^

The association of pyoderma gangrenosum with systemic diseases occurs in 50% of the cases.^3^ The main related conditions are ulcerative colitis, Crohn's disease, arthritis and hematologic malignancies, the latter present in 3.9% to 7% of patients. Among hematologic malignancies, AML, CML, MM, and asymptomatic monoclonal gammopathy of uncertain significance (MGUS) are the most frequently associated.^3^, ^6^, ^22^ The bullous form, which accounts for 50% of pyoderma gangrenosum cases seen in hematologic cancer, is often seen in AML, but also occurs in myelodysplastic syndrome (MDS), CML, polycythemia vera (PV), essential thrombocythemia, and myelofibrosis.^4^ The pustular form is more often associated with inflammatory bowel disease.^17^ The development of pyoderma gangrenosum in cases of hematologic malignancy confers a worse prognosis to the underlying disease.^23^

For the histopathological diagnosis, a spindle-shaped excision that includes the border and the base of the ulcer is preferable.^7^ Histopathology discloses the presence of a suppurative neutrophilic infiltrate in the dermis and subcutaneous tissue, accompanied by hemorrhage and necrosis. It is necessary to exclude infectious causes to attain the diagnosis, with staining for fungi and mycobacteria.^7^

Treatment always involves local care, with cleaning and appropriate dressings for each case. The care of the borders of lesions is important, where zinc oxide or petrolatum may be used.^23^ Adequate analgesia is also crucial.^18^

In mild cases, topical treatment with a high-potency corticosteroid such as clobetasol, a calcineurin inhibitor, or an intralesional corticosteroid may be used.^24^ For extensive cases, treatment with systemic corticosteroids (0.5 to 1 mg/kg/day) is indicated and, after improvement, weaning should be slow to prevent a recurrence. Cyclosporine at a dose of 4 mg/kg/day shows a rapid response and efficacy comparable to corticosteroids, but its use in cases associated with hematologic malignancies is controversial.^6^, ^18^ Other systemic options include pulse therapy with methylprednisolone, methotrexate, mycophenolate, colchicine, sulfasalazine, dapsone, minocycline, apremilast, and thalidomide.^18^

Immunobiologicals are also used in the treatment of pyoderma gangrenosum. The introduction of an immunobiological should be considered as an early option. In a randomized clinical trial, 69% of the patients treated with infliximab showed improvement and 21% experienced remission six weeks after infusion of the first dose.^25^ In addition to infliximab, adalimumab, etanercept, and ustekinumab are also used.^18^ In disease refractory to other alternatives, the use of intravenous immunoglobulin, cyclophosphamide or chlorambucil should be considered.

### Subcorneal pustulosis

Subcorneal pustular dermatosis or subcorneal pustulosis, is a rare dermatosis characterized by flaccid subcorneal pustules with a chronic course.

In its pathogenesis, the presence of cytokines related to neutrophil migration, such as TNF-α, IL-8, C5a, is identified in the more superficial layers of the epidermis.^26^

Lesions usually occur on the trunk and in intertriginous areas and are distributed in a serpiginous or annular pattern. The pustules are usually small and appear in outbreaks on normal or slightly erythematous skin. In larger pustules, the secretion collects in the lower portion of the lesion, an appearance known as a ‘half-and-half’ pustule. Over its course, there is the possibility of slight hyperpigmentation, following the resolution of the pustules.^26^

Subcorneal pustulosis is associated with hematologic diseases, especially IgA paraproteinemia. Other associations include IgA myeloma, aplastic anemia, lymphomas, CML, and polycythemia vera.^6^

On histopathological examination, a subcorneal neutrophilic abscess is present, with or without a mixed superficial perivascular infiltrate and absence of spongiosis.^3^

Treatment is carried out with dapsone, starting at a dose of 25 mg, with a target of 50 to 150 mg/day. The lowest dose needed to control symptoms should be maintained, and the monitoring of hematologic toxicity is mandatory.^26^ Other therapeutic options include phototherapy, topical corticosteroids, oral corticosteroids, and acitretin.^6^

### Neutrophilic eccrine hidradenitis

Neutrophilic eccrine hidradenitis is an uncommon neutrophilic dermatosis that was originally described in patients with AML treated with cytarabine and later identified in untreated cases of AML and CML. In cases associated with chemotherapy, it was considered the result of direct drug toxicity to the eccrine sweat gland.^4^ In a report of 51 cases of neutrophilic eccrine hidradenitis, 67% were AML cases that had received chemotherapy.^27^ Other associated conditions are chronic lymphocytic leukemia (CLL) B, CMML, Hodgkin's lymphoma (HL), and non-Hodgkin's lymphoma (NHL).^6^

**Figure 3:**
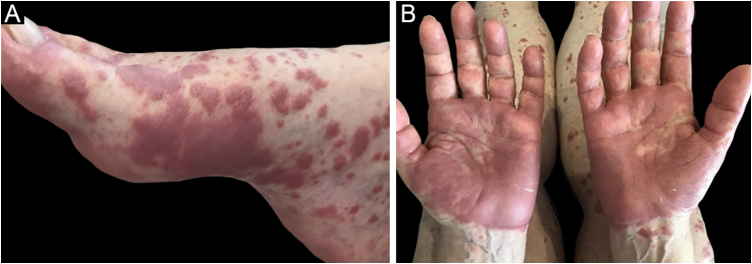
Neutrophilic eccrine hidradenitis in a patient with AML. Erythematous-violaceous, edematous, well-defined, very painful plaques affecting (A) the dorsum of the foot and (B) the palms.

The clinical picture is characterized by erythematous-edematous plaques that can be pruriginous or painful, dispersed on the hands, face, and extremities (Fig. 3). There is a possibility of fever, particularly in cases with associated neutropenia.^6^

Histopathology reveals a neutrophilic infiltrate in the eccrine sweat glands and ducts. There may be intraductal abscess formation, syringo-squamous metaplasia of the sweat glands, and fibrosis of the adjacent dermis.^27^

Some cases of neutrophilic eccrine hidradenitis resolve spontaneously; however, according to the authors' experience, the patient's quality of life is greatly impacted and disease management can be challenging. Several treatments are recommended, such as topical or systemic corticosteroids and dapsone.^4^

### Eosinophilic dermatoses

Eosinophilic dermatoses are part of the group of paraneoplastic dermatoses in patients with hematologic malignancies and are clinically characterized by pruriginous papules, vesicles, bullae, or nodules, possibly painful, similar to a reaction to an insect bite, dispersed throughout the body. Lesions may develop on an erythematoedematous, urticarial base.^28^

Eosinophilic dermatoses are mainly associated with B CLL and other B lymphoproliferative diseases, such as mantle cell lymphoma, acute lymphoblastic leukemia, and large B-cell lymphoma, but also with T lymphomas and acute leukemias.^6^, ^28^

The pathogenesis includes increased production of IL-4 and IL5 triggered by an insect bite, drug, or virus exposure, which leads to an altered immune response with a predominance of eosinophilic proliferation.^29^ Another possibility is that the hematologic disease or its treatment causes a predominance of the T-helper 2 (Th2) repertoire, which would favor the recruitment of T cells to the skin and the activation of eosinophils in response to environmental antigens.^6^

**Figure 4:**
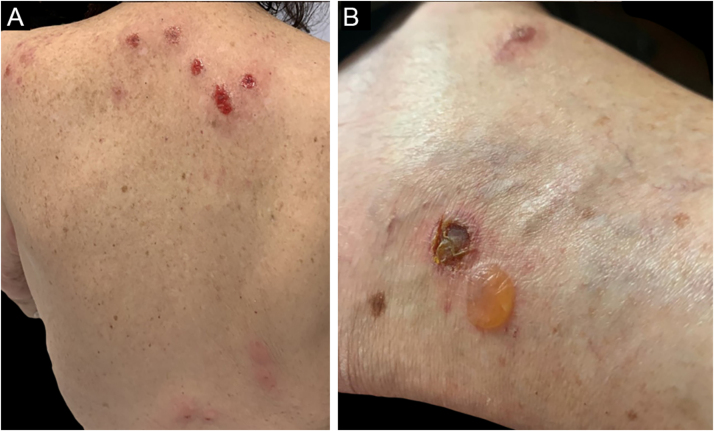
Eosinophilic dermatosis in patients with CLL. (A) Erythematous-edematous plaques on the back with ulceration on the upper back resulting from the act of scratching due to intense pruritus. (B) Bullous form with a lesion similar to bullous pemphigoid.

Three clinical forms have been described: bullous, characterized by vesiculo-bullae similar to bullous pemphigoid (Fig. 4); insect-bite reaction type, characterized by urticarial papules and plaques, and cellulitis-like, characterized by plaques and nodules similar to Wells syndrome.^6^ Lesions usually occur months or years after the hematologic diagnosis, but in rare cases, they precede the hematologic picture.^30^

The following criteria are used in the diagnosis: 1) Pruriginous papules, nodules, or vesiculo-bullae refractory to conventional treatments; 2) Presence of a superficial and deep lymphohistiocytic infiltrate, rich in eosinophils; 3) Exclusion of other causes of tissue eosinophilia; 4) Diagnosis of hematologic malignancy.^31^

Histopathology demonstrates a eosinophil-rich perivascular and periadnexal dermal lymphocytic inflammatory infiltrate, which may extend to the subcutaneous tissue. Flame figures may be present which are also found in Wells syndrome, eosinophilic cellulitis, bullous pemphigoid, pharmacopeias, and other conditions with an accumulation of eosinophils in the dermis.^32^

The treatment of eosinophilic dermatoses includes topical corticosteroids, phototherapy, systemic corticosteroids, dapsone, immunosuppressants such as methotrexate and azathioprine, lenalidomide, and treatment with dupilumab has also been reported. Concomitant treatment of the underlying hematologic disease may contribute to improvement of the clinical picture.^28^, ^33^

### Pruritus

Pruritus is defined as an unpleasant sensation that causes the urge to scratch. It is considered one of the symptoms that most often disturbs patients and alters their quality of life and is considered debilitating in many cases.^34^, ^35^, ^36^ It may be the first symptom of hematologic systemic involvement and precedes the diagnosis of the underlying disease in more than 50% of the cases,^34^, ^35^, ^36^ with a latency of months to up to 38 years.^34^, ^36^, ^37^

The pathogenesis of pruritus is complex; studies suggest the activation of a distinct pathway of unmyelinated C fibers via the spinothalamic tract to different parts of the brain. This pathway is influenced by a variety of inflammatory cells through complex interactions with the release of cytokines, proteases, and neuropeptides.^35^, ^37^

Myeloproliferative neoplasms, such as CML, polycythemia vera, primary myelofibrosis, and essential thrombocythemia have pruritus as the second most reported symptom by patients in 56% of cases, second only to fatigue. Both Hodgkin's and non-Hodgkin's lymphomas are also associated with pruritus, as they are primary MDS.^34^ Nevertheless, hematologic causes are listed in only 2% of all cases.^35^, ^37^

Pruritus is found in up to 69% of PV patients according to different studies.^34^, ^38^ It usually appears as aquagenic pruritus, that is, an itching, stinging, burning, or prickling sensation after contact with water on the skin, which lasts a few minutes, without forming any lesions.^38^ About a quarter of patients report a debilitating condition that interferes with daily activities.^34^, ^36^ The pathogenesis of aquagenic pruritus in PV involves an increase in mononuclear and eosinophil cells in the dermal papilla, edema, vasodilation, increased fibrinolytic activity, and activation of acetylcholinesterase. The JAK2V617 F mutation, found in PV, promotes basophil activation and hypersensitivity.^34^

Primary myelofibrosis is a disease characterized by myeloproliferation, reactive bone marrow fibrosis, osteosclerosis, angiogenesis, extramedullary hematopoiesis and alteration in cytokine expression. Pruritus is described in up to 16% of cases and was associated in one study with leukocytosis. Of the total number of patients with myelofibrosis-related pruritus, about 50% have aquagenic pruritus.^34^, ^37^, ^39^, ^40^

Pruritus is present in almost 40% of patients with Hodgkin's lymphoma and is severe in about 6% of them. It can precede the disease by up to four years and usually occurs in the legs and at night. Transient hyperhidrosis before the onset of pruritus between the fingers and on the palms has also been described. The pathogenesis is linked to Th2 response, production of IL-10, IL-4, IL-5, IgE and hypereosinophilia. On the other hand, pruritus is a rare finding in NHL, except for cutaneous T lymphomas, which will not be addressed in this review.

In suspected pruritus caused by hematologic neoplastic diseases, the initial investigation should include a detailed history and general physical examination. The mentioned initial screening tests are the complete blood count, erythrocyte sedimentation rate (ESR), LDH, chest X-ray and abdominal ultrasonography (US). When PV is suspected, screening for the JAK2V617 mutation is critical.^35^ According to the results of the initial evaluation, the investigation should be expanded together with the hematologist encompassing a bone marrow biopsy and specific imaging exams as required for each case.

The treatment of pruritus related to hematologic diseases is a challenge. The treatment of choice aimed at the underlying disease is always the best solution, although the effectiveness is not directly proportional. It is questioned that it occurs because the symptom is not directly related to the presence of abnormal cells, but rather to the immunological reaction that accompanies the disease.^35^, ^37^ Symptomatic measures to control pruritus are necessary in many cases, due to the significant decrease in quality of life. Antihistamines, which are often prescribed, do not relieve symptoms in most patients and, among these, the first-generation ones lead to better results when compared to the second-generation ones, due to the sedation they cause. Serotonin reuptake inhibitors such as paroxetine, the anticonvulsants gabapentin and pregabalin, and opioid receptor antagonists such as naltrexone are relatively effective in controlling this symptom. Phototherapy is also effective in many cases.^34^, ^35^, ^37^, ^39^ Aspirin has shown moderate results in cases of PV, and recent studies have encouraged the use of JAK and mTOR inhibitors in pruritus related to PV and primary myelofibrosis.^40^ Corticosteroids can be used to control pruritus in intractable lymphomas.^35^ Recently, aprepitant, a neurokin-1 receptor antagonist, which is already used in pruritus of cutaneous lymphomas and solid tumors, has been mentioned in the treatment of pruritus of a few cases of hematologic diseases with good results.^41^ The management of pruritus in patients receiving palliative care may require other options, such as thalidomide, which is avoided as a conventional use in cancer patients because of the range of side effects, particularly peripheral neuropathy, which overlaps with other drugs already being used.^35^

### Prurigo

Chronic prurigo (CP) is characterized by intense pruritus, associated with numerous symmetrically distributed papules, hyperkeratotic or excoriated nodules, and scars, mainly on the lower limbs and trunk and in areas accessible to scratching.^42^, ^43^ The skin between the lesions is preserved.^44^ CP as a sign of paraneoplastic disease is seen in leukemia and Hodgkin's disease,^43^ and is rare in non-Hodgkin's lymphoma.^45^

Patients with chronic prurigo should undergo a detailed medical history, and careful physical examination, including palpation of lymph nodes, liver, and spleen.^46^ Complementary tests should be performed to rule out underlying comorbidities and/or malignancies, according to the history and physical examination findings.^42^ Frequent clinical follow-up is suggested, with the repetition of this investigation every three to six months to attain an early definitive diagnosis of possible underlying malignant disease.^46^

The initial treatment of chronic paraneoplastic pruritus with topical corticosteroids, phototherapy, and antihistamines has shown to be ineffective.^45^ The diagnosis and treatment of the underlying disease induce a significant improvement of the condition, with control of skin lesions and pruritus.^45^

### Vasculitis

The term cutaneous vasculitis includes a wide variety of clinical entities that have as a common feature histopathological findings with perivascular inflammation and blood vessel damage. There is no single consensus on the best way to divide the vasculitis. In this review, the authors will address how hematologic malignancies relate to cutaneous small vessel vasculitis (CSVV). They will also address two other vasculitis with distinct cutaneous clinical findings or laboratory characteristics: polyarteritis nodosa (PAN) and erythema elevatum diutinum (EED).

### Cutaneous small vessel vasculitis

CSVV has multiple etiologies, being associated with neoplasms in 3.8%‒8% of cases, mainly lymphoproliferative disorders.^3^, ^4^, ^6^ The most commonly found hematologic disorders are MDS, but there are also reports of AML, CML, myelofibrosis, PV, and essential thrombocythemia.^3^, ^4^, ^6^, ^47^ Patients with paraneoplastic vasculitis are often older than patients with other types of vasculitis, and classic precipitating factors, such as infections or vasculitis, are typically not found in the patient history.^48^, ^49^

The mechanism by which paraneoplastic vasculitis occur is not fully elucidated, but a combination of factors is believed to be responsible: abnormal clearance of immune complexes, binding of non-specific antibodies on vascular walls, and dysregulated production of immunoglobulins.^48^ In hematologic cases, it is suggested that increased blood viscosity contributes to reducing the clearance of immune complexes in the small vessels of the dermis.^6^

The most common clinical presentation is palpable purpura that does not disappear with digital pressure, usually in the distal lower limbs, but also affects the upper limbs and the trunk. The lesions are painful or pruriginous.^48^, ^50^ Rarely, lesions coalesce into plaques or form hemorrhagic bullae.^4^ Vasculitis associated with hematologic malignancies have a longer duration than non-neoplastic ones.^6^ Constitutional symptoms may be present, such as weight loss and anorexia, arthritis and arthralgia, polyneuropathy, and abdominal pain, and are more common than in patients with non-neoplastic vasculitis.^48^ In most cases, the vasculitis occurs concomitantly with the diagnosis of the underlying neoplasm, and it may precede the diagnosis or even occur at a subsequent moment.^51^

Histopathological findings include perivascular neutrophil infiltration, fibrinoid necrosis of the vascular walls, the presence of nuclear dust, and erythrodiapedesis.^4^, ^6^, ^48^ Histopathology is indistinguishable from non-paraneoplastic vasculitis.^49^ However, the finding of neoplastic cells in the inflammatory infiltrate suggests the diagnosis of leukemic vasculitis, a rare presentation of leukemia cutis, associated with a worse prognosis.^6^ Positive rheumatoid factors and cryoglobulins can be found, usually at low titers and not diagnostically significant for other entities.^48^

The treatment of vasculitis depends on the treatment of the associated neoplasm, as well as its prognosis.^48^ In refractory cases, systemic corticosteroid therapy can be employed, with paraneoplastic cases being more resistant to treatment.^4^, ^6^ Improvement of vasculitis with chemotherapy to treat the hematologic disorder, with subsequent recurrence of skin lesions, is a sign of neoplastic recurrence.^6^

### Polyarteritis nodosa

PAN is a type of vasculitis that affects small and medium-sized vessels of multiple organs, including the skin. Among hematologic malignancies, PAN is most often associated with hairy cell leukemia.^4^ Among myeloid malignancies, the most common are MDS and CMML.^47^

The kidney is the most frequently affected organ in PAN. Renal impairment occurs in 70%‒80% of patients and manifests as renal failure, hypertension, proteinuria, or even hematuria. Spontaneous and bilateral perirenal hemorrhages seem to be more common in patients with MDS, as well as hemorrhages in other organs. This fact is likely caused by the simultaneous presence of thrombocytopenia and other coagulation disorders in combination with vascular abnormalities.^47^

In the skin, paraneoplastic PAN presents with the same characteristics as the classic form: subcutaneous nodules, palpable purpura, livedo reticularis or racemosa, ulcerations and bullae. The lower limbs are preferentially affected, followed by the upper limbs and trunk. Other systemic manifestations include fever, asymmetric polyarthritis, abdominal pain, and peripheral neuropathies.^3^, ^4^

The typical histopathological finding is necrotizing vasculitis of medium-sized vessels, found in the skin or other affected organs. P-ANCA (anti-neutrophil cytoplasmic antibody) is found in up to 20% of cases of cutaneous PAN, being generally absent in systemic vasculitis.^4^

Treatment is based on the control of the underlying disease, high-dose systemic corticosteroid therapy with a possible association with other immunosuppressants such as methotrexate or cyclophosphamide. The development of vasculitis in patients with MDS is associated with a worse prognosis of the underlying disease.^3^

### Erythema elevatum diutinum

Erythema elevatum diutinum (EED) is a chronic and rare form of localized leukocytoclastic vasculitis, initially described by Hutchinson in 1888. In the initial description, it was divided into two forms, the first being more common in elderly men and the second being found in young women with associated rheumatologic diseases. Both forms are currently described as the same entity and the term ‘diutinum’ means “long-lasting” and refers to the long and recurrent characteristic of the disease.^52^

Clinical lesions present as erythematous-violaceous papules, nodules, or plaques with a predilection for limb extensor surfaces.^4^ Cutaneous involvement occurs, in order of prevalence, on the dorsum of the hands, elbows, legs, knees, and feet, and eventually the palmoplantar region.^52^, ^53^ Occasionally, the lesions resemble vesicles, hemorrhagic nodules, or ulcerations.^53^ They are usually asymptomatic but may present with pruritus, pain, or even underlying arthralgia.^52^ Older lesions with partial resolution are yellowish or brownish in color, resembling a xanthoma.^53^

Histopathology of early lesions discloses leukocytoclastic vasculitis that progresses to perivascular fibrosis, with extracellular lipid deposition in the advanced stages of the disease.^52^ Wedge-shaped polymorphonuclear infiltrates occur with fibrin deposition on the superficial and mid-dermis.^53^ The absence of polymorphonuclear leukocytes should suggest another diagnosis.^53^

The pathophysiology involves the deposition of immune complexes in small vessels, leading to an inflammatory response with leukocyte recruitment.^52^ The most common associations are with infectious conditions such as HIV, often co-infected with viral hepatitis, cytomegalovirus (CMV), or other opportunistic infections, in addition to tuberculosis and recurrent streptococcal infection.^53^ In 30% of cases, it is associated with autoimmune diseases such as rheumatoid arthritis, systemic lupus erythematosus, or celiac disease.^52^ It may also be related to several hematologic malignancies, particularly IgA monoclonal gammopathies^54^ and MDS. More rarely, it is associated with MM, CML, and non-Hodgkin's lymphoma.^6^, ^55^

The dermatologic presentation may precede the hematologic picture by many years.^55^ Spontaneous regression occurs on average five to ten years after the diagnosis.^53^ However, there have been reports of longer periods, such as 25 to 39 years.^55^ The treatment of choice is oral dapsone;^6^ in addition, control of the underlying disease is related to control of EED in some cases.^55^ Treatment with intralesional medications or even surgical excision is only recommended in localized cases.

### Paraneoplastic pemphigus

Paraneoplastic pemphigus (PNP) is a rare variant of pemphigus, initially described by Anhalt et al. in 1990, who differentiated this entity from pemphigus vulgaris, when they demonstrated an association with malignant neoplasms and the presence of antibodies against desmoplakin I and BP 230 antigen.^56^ It is a rare disease, with about 500 cases described in the literature, and occurs in any age group, including children, but the mean age is 64.7 years.^57^, ^58^ Some studies show it seems more likely to affect the male sex, while others show no gender predilection.^59^, ^60^, ^61^ Due to its distinct clinical, histopathological, and immunological characteristics and its association with higher mortality than other types of pemphigus, the dermatologist must be attentive to the diagnosis of this disease.

The association with neoplasms or hematologic disorders is found in more than 84% of cases. Lymphoproliferative diseases are the most frequently associated, with non-Hodgkin's lymphoma being the most common one (38.6% of all cases of PNP), followed by CLL (18.4%), Castleman's disease (18.4%), thymomas (5.5%), Waldenström’s macroglobulinemia (1.2%), Hodgkin's lymphoma (0.6%), and monoclonal gammopathy (0.6%).^57^ The neoplasm is usually diagnosed before the PNP manifestation. However, 30% of cases have the cutaneous manifestation before the diagnosis of the occult neoplasm.^62^

The main clinical feature of PNP is stomatitis, erosions that progress to severe refractory oral and conjunctival ulcerations that affect the entire surface of the oropharynx, extending to the vermilion of the lips.^63^

Two-thirds of the patients have severe pseudomembranous conjunctivitis, which can lead to scarring and obliteration of the conjunctival fornices. Lesions in the nasopharynx, esophagus, vaginal, labial, penile, and perianal mucosa are also found.^60^

**Figure 5:**
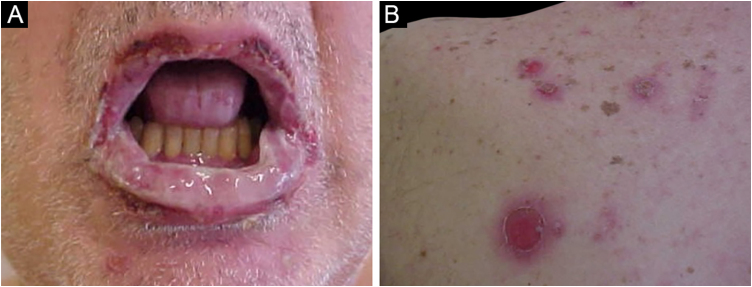
Paraneoplastic pemphigus. (A) Erosions on the entire labial vermilion surface. (B) Ulcerations on the upper back, similar to pemphigus vulgaris lesions.

The cutaneous lesions usually appear after the mucosal lesions and are polymorphic: erythematous macules, flaccid bullae, pemphigus vulgaris-like erosions (Fig. 5), tense bullae, lichenoid eruptions, and erythema multiforme-like lesions. The picture is usually diffuse with a predilection for the trunk and proximal extremities, affecting more than 50% of the integument or even causing erythroderma.^60^ The head and face are usually spared. However, in patients receiving radiotherapy, the lesions may initially concentrate at the irradiated site with further dissemination, and a potential misdiagnosis of radiation dermatitis.^64^ Palmoplantar and nail involvement with paronychia is usually present.^60^ The presence of erythema multiforme-like lesions on palms and soles is useful to differentiate from pemphigus vulgaris, in which palmoplantar lesions are uncommon.

Lichenoid lesions predominate in children and in East Asian populations and may have clinical and histopathological characteristics that are similar to erythema multiforme or graft-versus-host disease (GvHD).^59^, ^63^

Some patients develop bronchiolitis obliterans, which can lead to fatal respiratory failure and is a major cause of death in patients with PNP.^57^, ^65^

The pathophysiology of PNP is more complex than that of pemphigus vulgaris and is yet to be defined; humoral and cell-mediated autoimmune responses are involved in its onset. Theories about the pathogenesis of PNP include:

• Emergence of an immune response against neoplastic antigens with cross-reaction to epithelial antigens
• Production of antibodies by tumor components against epidermal antigens
• Dysregulation of the immune system, with an exaggerated synthesis of cytokines such as IL-6, leading to differentiation of B cells, the levels of which are elevated in PNP.^66^

The antibodies that are most commonly found in PNP are those against envoplakin, periplakin and α2-macroglobulin-like protein 1. IgG antibodies against desmoglein 3 or 1 are rarely found unlike in pemphigus vulgaris. Other autoantibodies found are those against desmocollin or BP180/BP230. For the serological diagnosis, plakin-rich substrates should be used for indirect immunofluorescence, such as mouse bladder, instead of human skin or monkey esophagus.^6^

Due to the assortment of clinical manifestations, the variability of histopathological findings is high. In many stomatitis lesions, only inflammation, necrosis and suprabasal acantholysis are found in the perilesional mucosa. Intact bullae show suprabasal acantholysis; however, unlike other types of pemphigus, there is significant inflammatory infiltrate even in early lesions. Non-bullous lesions may show keratinocyte necrosis, lymphocytic infiltrate in the epidermis, vacuolar changes, and interface dermatitis. Immunofluorescence shows intraepithelial anti-IgG antibodies, although false negative results are more common in PNP than in pemphigus vulgaris, requiring multiple biopsies for the diagnosis. A small number of cases also have positive IgG on the basement membrane.^66^

Anhalt proposed a redefinition of the original diagnostic criteria in 2004, where he considers as the minimum criteria for PNP diagnoses with a high degree of confidence: (1) Erosive and progressive stomatitis, with preferential involvement of the tongue; (2) Histopathological findings of acantholysis, lichenoid alterations, or interface dermatitis; (3) Demonstration of antiplakin autoantibodies, which are the key serological marker of PNP; (4) Demonstration of superjacent lymphoproliferative disease. In cases where the neoplasm is not present before the diagnosis, it is usually associated with Castleman disease, abdominal lymphoma, thymoma, or retroperitoneal sarcomas, detected by computed tomography (CT) scans of the chest, abdomen, and pelvis.^66^

The differential diagnosis will depend on the predominant clinical lesions. In patients with exclusive oral involvement, the differential diagnoses include pemphigus vulgaris, oral lichen planus, and major aphthous stomatitis. In patients with predominant lichenoid lesions, one must differentiate between lichen planus and GvHD. Patients with cutaneous and oral lesions may pose a difficult diagnosis between pemphigus vulgaris and PNP. Some characteristics differentiate these two diseases: pemphigus vulgaris shows discreet, superficial oral involvement, with areas of healthy mucosa, while PNP presents as a diffuse, deep and often necrotic condition. Pemphigus vulgaris rarely affects mucous membranes other than the oral mucosa. While pemphigus vulgaris is commonly found on the scalp and spares the palmoplantar areas, PNP affects the palms and soles and usually spares the scalp. Finally, Nikolky sign is positive in pemphigus vulgaris and negative in PNP.^67^

A small number of patients with recurrent erythema multiforme have positive antibodies against desmoplakin and will have positive indirect immunofluorescence on bladder epithelium. However, unlike PNP cases, these patients won’t have (1) underlying malignancy; (2) antibodies against plakins more specific for PNP, such as envoplakin and periplakin, and (3) the antibodies are transient and only detectable during episodes of active disease.^66^

The prognosis of PNP depends on the associated neoplasm. After the autoimmune disease onset, even with treatment and cure of the hematologic disease, there is a possibility that PNP will progress. In patients with benign neoplasms or localized tumors, tumor excision can substantially improve or even lead to remission of pemphigus. In patients with tumors treated surgically, about 50% go into remission. This remission occurs within one to two years after surgery, and immunosuppressive therapy is typically used during this period.^67^

There are no randomized clinical trials (RCTs) on treatment of PNP due to the rarity of the disease, but regimens suggested in the literature include the combination of prednisone, cyclosporine, and cyclophosphamide with tapering doses as clinical improvement occurs.^64^ Other therapeutic options such as dapsone, azathioprine, plasmapheresis, photopheresis, and immunoglobulins have been used, but none showed relevant efficacy. Skin lesions respond more quickly to therapy, whereas stomatitis is refractory to treatment.^66^

Patients with malignant neoplasms have worse responses to immunosuppressive therapies and cases associated with non-Hodgkin's lymphoma seem to have a worse prognosis than patients with CLL. Almost all patients with LNH or CLL will die within three months to two years after the diagnosis. Systemic corticosteroid therapy may lead to partial improvement of lesions but not their resolution.^6^

Despite the good response to rituximab in patients with pemphigus vulgaris and pemphigus foliaceus, the response in PNP is much less consistent. Complete remission is rare, and there are cases of PNP described in patients who received rituximab as part of the chemotherapy for the malignancy.^58^

Alemtuzumab, a monoclonal antibody against CD52 expressed on T and B lymphocytes, has shown to be promising in the treatment of some cases related to hematologic malignancies, but has not prevented the progression of pulmonary disease and is associated with significant immunosuppression after treatment.^58^

### Acquired ichthyosis

Acquired ichthyosis (AI), unlike ichthyosis vulgaris (IV) manifests more frequently in adulthood, although the clinical picture is similar. The clinical manifestations include rhomboid scales, which range from rough skin to lamellar desquamation resembling fish scales, with free borders,^3^, ^49^, ^68^ hypochromic to gray or brownish color, with a diameter ranging from less than 1 mm to more than 1 cm. It affects mainly the trunk and limbs, is more pronounced on the extensor surfaces, generally spares flexures, and affects the lower extremities to a greater extent than the upper ones. Skin lesions cause pruritus, which may also be present in normal-appearing areas; excoriations secondary to pruritus are often found.^49^, ^68^ It occurs when the cornification process is interrupted, resulting in hyperkeratosis, desquamation, and skin barrier function abnormalities. The pathogenesis is unknown but reduced dermal lipid synthesis and cholesterol deficiency are frequently seen in these patients and are likely to be involved in the pathogenesis.^3^, ^68^

The clinical and histopathological aspects are not enough to differentiate IV from AI, and a detailed clinical history is crucial. Essentially, all hereditary forms of ichthyosis will present clinically before the age of 13 years; in general, there is a positive family history and early age of onset, favoring the diagnosis of IV in these cases.^49^, ^68^

Histopathology reveals an increase in the stratum corneum, a reduced granular layer and a spinous layer with normal thickness. The absence of an inflammatory infiltrate in the dermis is among the typical histopathological findings. Epidermal thinning has been documented, as well as the presence of a mild perivascular lymphohistiocytic infiltrate in the papillary dermis, with no evidence of vasculitis.^49^, ^68^, ^69^

After the diagnosis of AI has been established, the patient should be evaluated for a possible triggering factor. AI can be due to malignant disease, non-malignant disease, or a drug reaction. If related to systemic disease, the cutaneous manifestation occurs before or after its identification. The AI intensity ​​may be associated with the severity or recurrence of the internal cause.^68^

The association between adult AI and malignancy is a frequent one, including Hodgkin's disease, non-Hodgkin's lymphoma, cutaneous T-cell lymphoma (CTCL), leiomyosarcoma, mycosis fungoides, MM, Kaposi's sarcoma, and, rarely, solid tumors, such as carcinoma of the ovary, breast, lung and uterine cervix. The most commonly reported malignancy in AI is Hodgkin's disease. However, there are other potential causes of acquired ichthyosis, including malnutrition, hypothyroidism, sarcoidosis, and drugs such as nicotinic acid and clofazimine.^49^, ^68^

The differential diagnoses include IV and Refsum disease. Refsum disease should be suspected in cases of ichthyosis that present from adolescence to the 3^rd^ decade of life and are associated with neurological findings such as decreased visual acuity, sensory neuropathy, and ataxia.^68^

Skin hydration and keratolytic agents are useful as treatment. Ichthyosis typically regresses once the underlying disease goes into remission.^49^, ^68^

### Erythema nodosum

Erythema nodosum (EN) is the most common form of panniculitis and is characterized by painful erythematous nodules, mainly affecting the lower limbs. It is more common in women and has a peak incidence in the population aged 20 to 30 years.^70^, ^71^

The clinical course of EN is characterized by acute onset of painful, erythematous nodules and plaques measuring one to six cm in diameter. The lesions are bilateral and symmetrical, normally distributed in the distal lower extremities in the pretibial areas, although the lesions can also affect the ankles, thighs, and forearms.^70^ They are usually self-limiting, resolving in two to eight weeks, changing from a bright red discoloration to a yellow-brown or blue-green discoloration, similar to a hematoma. The coexistence of lesions at different stages of evolution can be observed in the same patient. The nodules regress without ulceration, scarring, or atrophy, and recurrence is not uncommon.^70^, ^71^

Skin lesions are often accompanied by systemic symptoms such as fever, malaise, headaches, gastrointestinal complaints (such as abdominal pain, vomiting, and diarrhea), coughing, lymphadenopathy, weight loss, and arthralgia, particularly in the ankles and knees. Some of these findings suggest EN is secondary to a systemic disease, and these findings are likely to provide important diagnostic clues.^70^

The pathogenesis of erythema nodosum is unknown, although it seems to be a hypersensitivity response to a variety of antigenic stimuli. It has also been proposed that TNF-α and IL1-β, released by myelomonocytic cells, may be involved.^70^, ^71^

The diagnosis is usually attained through the characteristic clinical picture. However, it must be confirmed by an incisional or deep excisional biopsy, containing a generous portion of subcutaneous fat. Once the pathological diagnosis is made, the real challenge is to identify the underlying etiology, if present, before considering it to be idiopathic.^4^, ^70^ Histopathology predominantly shows septal panniculitis and the absence of primary vasculitis.^4^

The main differential diagnoses are erythema induratum of Bazin, superficial thrombophlebitis, panniculitis-like T-cell lymphoma, and other forms of panniculitis. Histopathology is essential for a more accurate diagnosis.^70^

Although the etiology is mainly idiopathic, ruling out an underlying disease is critical before diagnosing erythema nodosum. Erythema nodosum can be the first sign of a systemic disease, such as infections, inflammatory diseases, neoplasms, and dug reactions. The most common identifiable causes are streptococcal infections, primary tuberculosis, sarcoidosis, Behcet's disease, inflammatory bowel disease, dug reactions, and pregnancy. According to the literature, 32% to 72% of EN cases remain of unknown etiology.^4^, ^70^

An underlying malignancy may be responsible for EN symptoms in patients with an unexplained picture and constitutional symptoms. Hematologic malignancies, such as leukemia and lymphoma, including AML, CML, and CMML, among others, are most often associated with EN lesions, and more rarely, with solid neoplasms. In some cases, EN lesions indicate disease recurrence. Patients with recurrent EN or poor response to conventional treatments should be investigated for an underlying malignancy.^4^, ^70^

The investigation for suspected diseases should be carried out with a careful clinical history and physical examination. Systemic symptoms or altered laboratory test results favor secondary EN. The initial laboratory investigation should include a complete blood count, ESR, C-reactive protein, antistreptolysin O titer, throat swab culture, screening for tuberculosis in endemic areas, and chest X-ray. A pregnancy test should be performed on all women of childbearing age.^70^, ^72^

The initial approach comprises symptomatic support through compression bandages and limb elevation, aiming at reducing edema and alleviating pain.^70^ Non-steroidal anti-inflammatory drugs (NSAIDs) can be used for pain control. The use of systemic corticosteroids, potassium iodide, colchicine, dapsone, and hydroxychloroquine has also been described.^4^, ^70^

Specific therapy should be dedicated to the underlying associated condition when it is identified.^4^, ^72^

### Plane xanthoma

Diffuse plane xanthoma (DPX) is a rare disease, which belongs to the group of non-Langerhans histiocytoses and affects mainly adults, of both sexes.^73^, ^74^ Most patients are normolipidemic, but it also occurs in cases of hyperlipoproteinemia types 2 to 4. The presence of cutaneous xanthoma with normal plasma lipids requires further investigation and continuous monitoring for an underlying hematologic condition.^49^, ^75^

It presents as irregular, diffuse, symmetrical, and asymptomatic yellow-orange plaques or macules, affecting any part of the body, predominantly the face, trunk and intertriginous areas, such as the axillae. It is usually accompanied or preceded by xanthelasma.^49^, ^73^, ^74^

Extracutaneous lesions can be observed in the oral cavity, eyes, tendons, aortic valve, muscles and gastrointestinal system.^73^

Diffuse plane xanthomas are described in association with MM, monoclonal gammopathies, and, less frequently, lymphoproliferative disorders.^73^, ^74^

The pathogenesis of DPX is not clearly defined. In cases where there is a correlation with gammopathies, some authors believe that paraprotein-lipoprotein complexes are deposited in the skin. Others suggest infiltration of leukemic cells with subsequent xanthomization.^73^, ^74^

Histopathologically, foam cells - macrophages with lipid droplets - are present and a variable number of Touton giant cells, lymphocytes, and foamy histiocytes can be seen in the upper dermis and occasionally show a perivascular location.^73^, ^74^

The differential diagnoses include hyperlipidemic xanthomas and necrobiotic xanthogranuloma.^73^, ^74^

The prognosis depends on the underlying condition and treatment should be directed at the underlying disease. Remission of the hematologic condition results in cutaneous improvement, with relapse being indicated by recurrence of the skin lesions.^49^, ^73^

### Necrobiotic xanthogranuloma

Necrobiotic xanthogranuloma (NXG) is an uncommon non-Langerhans cell histiocytosis affecting the skin and extracutaneous tissues, with a predilection for the periorbital region.^76^, ^77^ It may affect the eye, leading to proptosis, blepharoptosis, and ocular motility limitation.^77^ Internal organs may also be affected, including the gastrointestinal and respiratory tracts.^77^, ^78^, ^79^ Rare cases present with lesions on the trunk or extremities, without facial involvement.^80^

NXG lesions most often start in the periorbital region, progressing to the trunk and extremities.^77^, ^78^ This disease is characterized by multiple asymptomatic yellowish to reddish-brown indurated papules and nodules that slowly progress to large plaques involving the dermis and subcutaneous tissue.^77^ Ulceration, telangiectasias, atrophy, and pruritus may also be present and are most commonly observed in the periorbital region.^77^, ^78^

The median age of onset is the 6^th^ decade of life, with cases being reported in the age range of 17 to 85 years, with no sex predilection.^80^

In approximately 80% of patients, monoclonal gammopathy is identified, resulting from plasma cell dyscrasia or lymphoproliferative disorder.^76^, ^77^, ^78^ Only 10% of cases have been observed to progress to MM.^80^

Other reported associations include non-Hodgkin's lymphoma, Hodgkin's lymphoma, Waldenström’s macroglobulinemia, MDS, CLL, and amyloidosis.^77^, ^80^, ^81^ All patients should be investigated for hematologic and lymphoproliferative disorders.^77^

Multiple myeloma that develops in patients with NXG seems to have a relatively benign behavior, with 90% to 100% of patients having a survival of ten to 15 years.^80^ The pathogenesis of NXG is yet to be fully understood.^77^

NXG and plane xanthoma (PX) are two distinct forms of xanthoma; cases reporting the coexistence of NXG and XP at the same time are rare.^82^ If a new NXG lesion develops in a patient with PX, a hematologic disease assessment is strongly recommended.^82^

Thinned or normal epidermis, extensive areas of necrobiosis, and granulomatous infiltrate with foreign body giant cells, Touton giant cells, and foam cells may be seen on histopathological examination.^76^, ^77^ Although there is no specific histopathological pattern for NXG, these changes, associated with the characteristic clinical picture, favor this diagnosis.^76^, ^77^

Treatment options include topical and systemic corticosteroids, thalidomide, high-dose intravenous immunoglobulin (IVIg), chlorambucil, cyclophosphamide, fludarabine, rituximab, melphalan, infliximab, interferon-α, cladribine, hydroxychloroquine, azathioprine, methotrexate, laser therapy, radiation therapy, surgery, PUVA, plasmapheresis and extracorporeal photopheresis, with variable and unpredictable responses.^76^, ^77^, ^79^

Treatment of the monoclonal gammopathy with alkylating agents does not necessarily influence skin disease activity.^77^, ^78^

NXG has a chronic, progressive, and indolent course. The prognosis depends on the severity of the extracutaneous manifestations, the presence of hematologic neoplasms, and complications of the cutaneous lesions.^80^

### Scleromyxedema

Scleromyxedema is a rare disease that mainly affects adult individuals between 30 and 80 years of age, regardless of ethnicity or sex. It has been rarely reported in infants and young children.^83^, ^84^ The condition is often associated with monoclonal gammopathies and the disease progresses to definitive MM in less than 10% of cases. The association with other hematologic malignancies, such as Hodgkin's and non-Hodgkin's lymphoma, Waldenström's macroglobulinemia, and CMML or visceral carcinomas has been reported.^49^, ^83^

It manifests as a generalized eruption of, firm, waxy-looking, closely spaced, dome-shaped, or flat papules measuring 2 to 3 mm, affecting the hands, forearms, head, neck, trunk, and upper thighs.^49^, ^83^ The papules are often arranged in a linear array and the surrounding skin is shiny and indurated (sclerodermoid). Subcutaneous nodules are rarely present. The glabella is typically affected, with deep longitudinal grooves that result in characteristic leonine facies.^83^ Deep grooves are occasionally evident on the trunk or limbs, resulting in the “Shar Pei sign”. Erythema, edema, and brownish color occur in unaffected areas; pruritus is common. Eyebrows, axillary and pubic hair are occasionally sparse in these patients. Mucous membranes are spared. As the disease progresses, erythematous and infiltrated plaques appear, with consequent stiffening of the skin, sclerodactyly and decreased motility of the mouth and joints. Over the proximal interphalangeal joint, a plaque with a central depression and a raised border (due to skin thickening) can be seen and is referred to as the “doughnut sign”.^49^, ^83^

In patients with scleromyxedema, the most common extracutaneous manifestations are neurologic, rheumatologic, and cardiac abnormalities.^83^

The pathogenesis of scleromyxedema is unknown. The main hypothesis is that circulating cytokines, such as IL-1, TNF-αand TGF-β, which stimulate the synthesis of glycosaminoglycans and the proliferation of fibroblasts in the skin, may play a role in lesion development. The clinical remission of scleromyxedema after autologous hematopoietic stem-cell transplantation (HSCT) suggests that the bone marrow may be a source of these cytokines. Despite the frequent association with monoclonal gammopathy, paraprotein levels generally do not correlate with disease severity and progression, or response to treatment.^83^

The diagnosis of scleromyxedema is based on the following clinicopathological criteria:

• Generalized/diffuse papular and sclerodermoid eruption;
• Triad of mucin deposition (mainly consisting of hyaluronic acid in the upper and middle reticular dermis), fibroblast proliferation and fibrosis;
• Monoclonal gammopathy;
• Absence of thyroid disorder.^83^, ^85^, ^86^

The main disorders to be considered in the differential diagnosis are scleredema, localized scleroderma, systemic sclerosis, nephrogenic systemic fibrosis, and lichen myxedematosus.^83^, ^85^

The therapeutic options include interventions aimed at treating associated plasma cell dyscrasia.^85^, ^86^

Due to the rarity of the disease, there is no specific effective treatment. First-line therapy is intravenous immunoglobulin (IVIg) at a dose of 2 g/kg, divided over two to five consecutive days, administered monthly.^87^, ^88^ The mechanism by which IVIg acts in scleromyxedema is unclear, but should be considered, particularly in patients with deterioration of cutaneous symptoms or life-threatening internal organ involvement.^83^, ^84^, ^85^

When IVIg treatment is not indicated or when an insufficient response occurs, thalidomide (or lenalidomide) and systemic glucocorticoids are the next-line treatment options. Thalidomide and systemic glucocorticoids are administered alone or in combination with IVIg.^81^, ^85^

Other skin-targeting strategies, such as extracorporeal photopheresis, PUVA, electron beam therapy, topical corticosteroids, and retinoids have been tried with varying degrees of success.^83^, ^85^ Patients may benefit from interventions aimed at treating the associated plasma cell dyscrasia.^85^

## Conclusion

As reviewed in both articles (part I and part II), cutaneous manifestations precede, accompany, or are identified after the diagnosis of hematologic neoplasms. Thus, the identification of these entities and their possible relationship with a probable underlying disease has significant diagnostic value.

The importance of recognizing the dermatological diseases associated with hematologic neoplasms is evident, as the cutaneous involvement worsens the morbidity of the patient who requires specific dermatologic treatment. Moreover, the presence of the skin disease may worsen the prognosis of the underlying hematologic condition and possibly trigger the need for a change in the systemic therapeutic support.

Everything discussed in this review highlights the importance of knowing the cutaneous manifestations that accompany systemic conditions, whether for the simple care to be offered to the patient and consequent maintenance of quality of life, but mainly for the early warning of the existence of an occult neoplasm or the worsening of an existing one.

In cancer patients, comorbidities add up, and dermatologic differential diagnosis is a challenge. Neoplastic, inflammatory, and infectious etiologies should be considered, in addition to pharmacodermias. The cutaneous manifestations of hematologic diseases are extremely variable in morphology, distribution, and symptomatology. A skin biopsy and immunohistochemical analysis contribute substantially to the clinical diagnosis, as well as the microbiological data of tissue fragments sent to cultures for fungi and bacteria.

Few studies have depicted the skin diseases that accompany the hematologic patient and little is known about the mechanisms that lead to these conditions, being a broad and important field of study. It is also little known how much these diseases impact the quality of life of hematologic patients an to what extend they can be used as prognostic markers.

The management of these conditions is not simple and portrays the importance of each step being discussed in a multidisciplinary way. Conducting the dermatological intervention jointly with the hematologist is always mandatory. Among many facts that must be observed it is relevant how much the treatment of the underlying disease will resolve the dermatological condition, and how much the specific dermatologic therapy will positively or negatively impact the overall context.

The dermatologist should join the hematologic team aiming to clarify the possible diagnosis of the skin condition, plan the treatment together, maintain the follow-up and, above all, provide the best care to the patient. The hematologist should examine the patient's skin and request a dermatologic intervention for a better diagnostic management. In current medical care, the importance of comprehensive, multidisciplinary/collaborative, technically excellent and broadly humanized care is crucial.

## Financial support

None declared.

## Authors' contributions

Patricia Karla de Souza: Design of the study; article writing and critical review of important intellectual content; design of the study together with the co-authors; critical review of the literature; approval of the final version of the manuscript.

Rafael Oliveira Amorim: Article writing and critical review of important intellectual content; design of the study together with the co-authors; critical review of the literature; approval of the final version of the manuscript.

Letícia Siqueira Sousa: Article writing and critical review of important intellectual content; design of the study together with the co-authors; critical review of the literature; approval of the final version of the manuscript.

Mariana Dias Batista: Article writing and critical review of important intellectual content; design of the study together with the co-authors; critical review of the literature; approval of the final version of the manuscript.

## Conflicts of interest

None declared.

## References

[1] Silva J.A., Mesquita K.C., Igreja A.C.S.M., Lucas I.C.R.N., Freitas A.F., Oliveira S.M. (2013). Paraneoplastic cutaneous manifestations: concepts and updates. An Bras Dermatol..

[2] Caccavale S., Brancaccio G., Agozzino M., Vitiello P., Alfano R., Argenziano G. (2018). Obligate and facultative paraneoplastic dermatoses: an overview. Dermatol Pract Concept..

[3] Zappasodi P., Forno C., Corso A., Lazzarino M. (2006). Mucocutaneous paraneoplastic syndromes in hematologic malignancies. Int J Dermatol..

[4] Li A.W., Yin E.S., Stahl M., Kim T.K., Panse G., Zeidan A.M. (2017). The skin as a window to the blood: cutaneous manifestations of myeloid malignancies. Blood Rev..

[5] Wallach D., Vignon-Pennamen M.D. (2006). From acute febrile neutrophilic dermatosis to neutrophilic disease: forty years of clinical research. J Am Acad Dermatol..

[6] Maglie R., Genovese G., Solimani F., Guglielmo A., Pileri A., Portelli F. (2020). Immune-mediated dermatoses in patients with haematological malignancies: a comprehensive review. Am J Clin Dermatol..

[7] Marzano A.V., Borghi A., Wallach D., Cugno M. (2018). A comprehensive review of neutrophilic diseases. Clin Rev Allergy Immunol..

[8] Lepelletier C., Bouaziz J.D., Rybojad M., Bagot M., Georgin-Lavialle S., Vignon-Pennamen M.D. (2019). Neutrophilic dermatoses associated with myeloid malignancies. Am J Clin Dermatol..

[9] Sujobert P., Cuccuini W., Vignon-Pennamen D., Martin-Garcia N., Albertini A.F., Uzunov M. (2013). Evidence of differentiation in myeloid malignancies associated neutrophilic dermatosis: a fluorescent in situ hybridization study of 14 patients. J Invest Dermatol..

[10] Ravi V., Maloney N.J., Worswick S. (2019). Neutrophilic dermatoses as adverse effects of checkpoint inhibitors: a review. Dermatol Ther..

[11] Rochet N.M., Chavan R.N., Cappel M.A., Wada D.A., Gibson L.E. (2013). Sweet syndrome: clinical presentation, associations, and response to treatment in 77 patients. J Am Acad Dermatol..

[12] Raza S., Kirkland R.S., Patel A.A., Shortridge J.R., Freter C. (2013). Insight into Sweet’s syndrome and associated-malignancy: a review of the current literature. Int J Oncol..

[13] Weedon D. (2010). In: Weedon’s Skin Pathology.

[14] Alegría-Landa V., Rodríguez-Pinilla S.M., Santos-Briz A., Rodríguez-Peralto J.L., Alegre V., Cerroni L. (2017). Clinicopathologic, immunohistochemical, and molecular features of histiocytoid Sweet syndrome. JAMA Dermatol..

[15] Ghoufi L., Ortonne N., Ingen-Housz-Oro S., Barhoumi W., Begon E., Haioun C. (2016). Histiocytoid Sweet syndrome is more frequently associated with myelodysplastic syndromes than the classical neutrophilic variant. Medicine..

[16] Cohen P.R., Kurzrock R. (2002). Sweet’s syndrome. Am J Clin Dermatol..

[17] Ruocco E., Sangiuliano S., Gravina A., Miranda A., Nicoletti G. (2009). Pyoderma gangrenosum: an updated review. J Eur Acad Dermatol Venereol..

[18] George C., Deroide F., Rustin M. (2019). Pyoderma gangrenosum – a guide to diagnosis and management. Clin Med..

[19] Binus A.M., Qureshi A.A., Li V.W., Winterfield L.S. (2011). Pyoderma gangrenosum: a retrospective review of patient characteristics, comorbidities and therapy in 103 patients. Br J Dermatol..

[20] Xia F.D., Liu K., Lockwood S., Butler D., Tsiaras W.G., Joyce C. (2018). Risk of developing pyoderma gangrenosum after procedures in patients with a known history of pyoderma gangrenosum ‒ A retrospective analysis. J Am Acad Dermatol..

[21] Barbosa N.S., Tolkachjov S.N., el-Azhary R.A., Davis M.D.P., Camilleri M.J., McEvoy M.T. (2016). Clinical features, causes, treatments, and outcomes of peristomal pyoderma gangrenosum (PPG) in 44 patients: the Mayo Clinic experience, 1996 through 2013. J Am Acad Dermatol.

[22] Langan S.M., Groves R.W., Card T.R., Gulliford M.C. (2012). Incidence, mortality, and disease associations of pyoderma gangrenosum in the united kingdom: a retrospective cohort study. J Invest Dermatol..

[23] Ahronowitz I., Harp J., Shinkai K. (2012). Etiology and management of pyoderma gangrenosum. Am J Clin Dermatol..

[24] Miller J., Yentzer B.A., Clark A., Jorizzo J.L., Feldman S.R. (2010). Pyoderma gangrenosum: a review and update on new therapies. J Am Acad Dermatol..

[25] Brooklyn T.N. (2006). Infliximab for the treatment of pyoderma gangrenosum: a randomised, double blind, placebo controlled trial. Gut..

[26] Cheng S., Edmonds E., Ben-Gashir M., Yu R.C. (2008). Subcorneal pustular dermatosis: 50 years on. Clin Exp Dermatol..

[27] Bachmeyer C., Aractingi S. (2000). Neutrophilic eccrine hidradenitis. Clin Dermatol..

[28] Sato‐Sano M., Teixeira S.P., Vargas J.C., Baiocchi O.C.C.G., Enokihara M.M.S.S., Gomes E.E. (2019). Lenalidomide in the management of eosinophilic dermatosis of hematological malignancy. J Dermatol..

[29] Rodríguez-Lojo R., Almagro M., Piñeyro F., Pérez-Varela L., Fernández-Jorge B., Pozo J.D. (2010). Eosinophilic panniculitis and insect bite-like eruption in a patient with chronic lymphocytic leukaemia: a spectrum of the same entity. Dermatol Res Pract.

[30] Farber M.J., la Forgia S., Sahu J., Lee J.B. (2012). Eosinophilic dermatosis of hematologic malignancy. J Cut Pathol..

[31] Byrd J.A., Scherschun L., Chaffins M.L., Fivenson D.P. (2001). Eosinophilic dermatosis of myeloproliferative disease: characterization of a unique eruption in patients with hematologic disorders. Arch Dermatol..

[32] Lucas-Truyols S., Rodrigo-Nicolás B., Lloret-Ruiz C., Quecedo-Estébanez E. (2017). Dermatosis eosinofílicas asociadas a procesos hematológicos. Actas Dermo-Sifiliograf..

[33] Jin A., Pousti B.T., Savage K.T., Mollanazar N.K., Lee J.B., Hsu S. (2019). Eosinophilic dermatosis of hematologic malignancy responding to dupilumab in a patient with chronic lymphocytic leukemia. JAAD Case Reports.

[34] Welz-Kubiak K., Reszke R., Szepietowski J.C. (2019). Pruritus as a sign of systemic disease. Clin Dermatol..

[35] Millington G.W.M., Collins A., Lovell C.R., Leslie T.A., Yong A.S.W., Morgan J.D. (2018). British Association of Dermatologists’ guidelines for the investigation and management of generalized pruritus in adults without an underlying dermatosis, 2018. Br J Dermatol..

[36] Siegel F.P., Tauscher J., Petrides P.E. (2013). Aquagenic pruritus in polycythemia vera: characteristics and influence on quality of life in 441 patients. Am J Hematol..

[37] Ceesay M.M., Basu T.N., du Vivier A., Mufti G.J. (2019). Intractable pruritus in chronic myelomonocytic leukaemia and myelodysplastic syndromes: a case series. BMJ Case Rep..

[38] Lelonek E., Matusiak Ł, Wróbel T., Szepietowski J.C. (2018). Aquagenic pruritus in polycythemia vera: clinical characteristics. Acta Dermato-Venereol..

[39] Vaa B.E., Tefferi A., Gangat N., Pardanani A., Lasho T.L., Finke C.M. (2016). Pruritus in primary myelofibrosis: management options in the era of JAK inhibitors. Ann Hematol..

[40] Vaa B.E., Wolanskyj A.P., Roeker L., Pardanani A., Lasho T.L., Finke C.M. (2012). Pruritus in primary myelofibrosis: clinical and laboratory correlates. Am J Hematol..

[41] Pulido-Perez A., Carretero-Lopez F., Bergon-Sendin M., Nieto-Benito L.M., Romero-Jimenez R., Dorado-Herrero N. (2020). Aprepitant in refractory pruritus of systemic lymphoproliferative disorders. J Eur Acad Dermatol Venereol..

[42] Serra-García L., Riera-Monroig J., Riquelme-Mc Loughlin C., Daniel C.M.C. (2021). Chronic prurigo as a onset of Hodgkin’s lymphoma. Med Clín..

[43] Dumont S., Péchère M., Toutous Trellu L. (2018). Chronic prurigo: an unusual presentation of Hodgkin lymphoma. Case Rep Dermatol..

[44] Shelnitz L.S., Paller A.S. (1990). Hodgkin’s disease manifesting as prurigo nodularis. Pediatr Dermatol..

[45] Schweda K., Hainz M., Loquai C., Grabbe S., Saloga J., Tuettenberg A. (2015). Prurigo nodularis as index symptom of (non-Hodgkin) lymphoma: ultrasound as a helpful diagnostic tool in dermatological disorders of unknown origin. Int J Dermatol..

[46] Callen J.P., Bernardi D.M., Clark R.A.F., Weber D.A. (2000). Adult-onset recalcitrant eczema: a marker of noncutaneous lymphoma or leukemia. J Am Acad Dermatol..

[47] Oostvogels R., Petersen E.J., Chauffaille M.L., Abrahams A.C. (2012). Systemic vasculitis in myelodysplastic syndromes. Neth J Med..

[48] Loricera J., Calvo-Río V., Ortiz-Sanjuán F., González-López M.A., Fernández-Llaca H., Rueda-Gotor J. (2013). The spectrum of paraneoplastic cutaneous vasculitis in a defined population. Medicine..

[49] Wick M.R., Patterson J.W. (2019). Cutaneous paraneoplastic syndromes. Sem Diagn Pathol..

[50] Chen K.R., Carlson J.A. (2008). Clinical approach to cutaneous vasculitis. Am J Clin Dermatol..

[51] Bachmeyer C., Wetterwald E., Aractingi S. (2005). Cutaneous vasculitis in the course of hematologic malignancies. Dermatol..

[52] Doktor V., Hadi A., Hadi A., Phelps R., Goodheart H. (2019). Erythema elevatum diutinum: a case report and review of literature. Int J Dermatol..

[53] Gibson L.E., el-Azhary R.A. (2000). Erythema elevatum diutinum. Clin Dermatol..

[54] Yiannias J.A., El-Azhary R.A., Gibson L.E. (1992). Erythema elevatum diutinum: a clinical and histopathologic study of 13 patients. J Am Acad Dermatol..

[55] Momen S.E., Jorizzo J., Al-Niaimi F. (2014). Erythema elevatum diutinum: a review of presentation and treatment. J Eur Acad Dermatol Venereol..

[56] Anhalt G.J., Kim S., Stanley J.R., Korman N.J., Jabs D.A., Kory M. (1990). Paraneoplastic Pemphigus. An autoimmune mucocutaneous disease associated with neoplasia. An autoimmune mucocutaneous disease associated with neoplasia. N Eng J Med..

[57] Paolino G., Didona D., Magliulo G., Ianella G., Didona B., Mercuri S.R. (2017). Paraneoplastic emphigus: insight into the autoimmune pathogenesis, clinical features and therapy. Int J Mol Sci..

[58] Amber K.T., Valdebran M., Grando S.A. (2018). Paraneoplastic autoimmune multiorgan syndrome (PAMS): beyond the single phenotype of paraneoplastic pemphigus. Autoimmun Rev..

[59] Sehgal V.N., Srivastava G. (2009). Paraneoplastic pemphigus/paraneoplastic autoimmune multiorgan syndrome. Int J Dermatol..

[60] Kimyai-Asadi A., Jih M.H. (2001). Paraneoplastic pemphigus. Int J Dermatol..

[61] Ogawa H., Sakuma M., Morioka S., Kitamura K., Sasai Y., Imamura S. (1995). The incidence of internal malignancies in pemphigus and bullous pemphigoid in Japan. J Dermatol Sci..

[62] Didona D., Fania L., Didona B., Eming R., Hertl M., di Zenzo G. (2020). Paraneoplastic dermatoses: a brief general review and an extensive analysis of paraneoplastic pemphigus and paraneoplastic dermatomyositis. Int J Mol Sci..

[63] Kartan S., Shi V.Y., Clark A.K., Chan L.S. (2017). Paraneoplastic pemphigus and autoimmune blistering diseases associated with neoplasm: characteristics, diagnosis, associated neoplasms, proposed pathogenesis, treatment. Am J Clin Dermatol..

[64] Plumb R., Doolittle G.C. (1996). Paraneoplastic pemphigus in a patient with non-Hodgkin’s lymphoma. Am J Hematol.

[65] Nousari H.C., Deterding R., Wojtczack H., Aho S., Uitto J., Hashimoto T. (1999). The mechanism of respiratory failure in paraneoplastic pemphigus. N Engl J Med..

[66] Anhalt G.J. (2004). Paraneoplastic pemphigus. J Invest Dermatol Symp Proc..

[67] Czernik A., Camilleri M., Pittelkow M.R., Grando S.A. (2011). Paraneoplastic autoimmune multiorgan syndrome: 20 years after. Int J Dermatol..

[68] Patel N., Spencer L.A., English J.C., Zirwas M.J. (2006). Acquired ichthyosis. J Am Acad Dermatol..

[69] Saardi K.M., DeWitt C.A., Cardis M.A. (2020). Acquired ichthyosis in a middle-aged woman. JAMA Dermatol..

[70] Pérez-Garza D.M., Chavez-Alvarez S., Ocampo-Candiani J., Gomez-Flores M. (2021). Erythema nodosum: a practical approach and diagnostic algorithm. Am J Clinl Dermatol..

[71] Xu X., Liang G., Duan M., Zhang L. (2017). Acute myeloid leukemia presenting as erythema nodosum. Medicine..

[72] la Spina M., Russo G. (2007). Presentation of childhood acute myeloid leukemia with erythema nodosum. J Clin Oncol..

[73] Falk L., Dyall-Smith D., Stolz W., Coras-Stepanek B. (2019). Diffuse plane xanthoma developing in association with prior monoclonal gammopathy. BMJ Case Rep..

[74] da Silva D.M., Chacha J.J., Wiziack N de C., Takita L.C., Hayashi F.K. (2010). Diffuse plane idiopathic normolipemic xanthoma with hiperesplenism. An Bras Dermatol..

[75] Zak A., Zeman M., Slaby A., Vecka M. (2014). Xanthomas: clinical and pathophysiological relations. Biomed Papers..

[76] Dellatorre G., Miqueloto J.K. (2020). Necrobiotic xanthogranuloma. JAMA Dermatol..

[77] Miguel D., Lukacs J., Illing T., Elsner P. (2017). Treatment of necrobiotic xanthogranuloma ‒ a systematic review. J Eur Acad Dermatol Venereol..

[78] Giacalone S., Genovese G., Benzecry V., Berti E., Cusini M. (2021). Necrobiotic xanthogranuloma in IgG‐κ multiple myeloma. Br J Haematol..

[79] Portuguese A.J., Long T.H., Linenberger M. (2021). Necrobiotic xanthogranuloma. Mayo Clin Proceed..

[80] Geoloaica L.G., Pătraşcu V., Ciurea R.N. (2021). Necrobiotic xanthogranuloma ‒ case report and literature review. Curr Health Sci J..

[81] Means A., Marvin E.K., Anderson K.R., Lehman J.S., Hertel D. (2022). Necrobiotic xanthogranuloma with type 1 cryoglobulinemia mimicking necrobiosis lipoidica in a young woman with myeloma. JAAD Case Rep..

[82] Kim J.G., Kim H.R., You M.H., Shin D.H., Choi J.S., Bae Y.K. (2020). Necrobiotic xanthogranuloma coexists with diffuse normolipidemic plane xanthoma and multiple myeloma. Ann Dermatol..

[83] Knobler R., Moinzadeh P., Hunzelmann N., Kreuter A., Cozzio A., Mouthon L. (2017). European dermatology forum S1-guideline on the diagnosis and treatment of sclerosing diseases of the skin, Part 2: scleromyxedema, scleredema and nephrogenic systemic fibrosis. J Eur Acad Dermatol Venereol..

[84] Heymann W.R. (2007). Scleromyxedema. J Am Acad Dermatol..

[85] Rongioletti F., Merlo G., Cinotti E., Fausti V., Cozzani E., Cribier B. (2013). Scleromyxedema: a multicenter study of characteristics, comorbidities, course, and therapy in 30 patients. J Am Acad Dermatol..

[86] Bhutani M., Shahid Z., Schnebelen A., Alapat D., Usmani S.Z. (2016). Cutaneous manifestations of multiple myeloma and other plasma cell proliferative disorders. Semin Oncol..

[87] Kim S., Park T.H., Lee S.M., Kim Y.H., Cho M.K., Whang K.U. (2020). Scleromyxedema with multiple systemic involvement: Successful treatment with intravenous immunoglobulin. Dermatol Ther..

[88] Haber R., Bachour J., el Gemayel M. (2020). Scleromyxedema treatment: a systematic review and update. Int J Dermatol..

